# TM4 of the glutamate transporter GLT-1 experiences substrate-induced motion during the transport cycle

**DOI:** 10.1038/srep34522

**Published:** 2016-10-04

**Authors:** Xiuliang Rong, Feng Tan, Xiaojuan Wu, Xiuping Zhang, Lingli Lu, Xiaoming Zou, Shaogang Qu

**Affiliations:** 1 Department of Blood Transfusion, The Fifth Affiliated Hospital, Southern Medical University, Guangzhou, Guangdong 510900, China; 2 Department of Immunology, School of Basic Medical Sciences, Southern Medical University, Guangzhou, Guangdong 510515, China; 3 Department of Genetic Metabolism, Children’s Hospital, Maternal and Child Health Hospital of Guangxi Zhuang Autonomous Region, Nanning, Guangxi 530003, China; 4 Department of Neurology, Foshan Hospital of Traditional Chinese Medicine, Guangzhou University of Chinese Medicine, Foshan, Guangdong 528000, China; 5 Teaching Center of Experimental Medicine, School of Basic Medical Sciences, Southern Medical University, Guangzhou, Guangdong 510515, China; 6 Department of Thoracic Cardiovascular Surgical, The Fifth Affiliated Hospital, Southern Medical University, Guangzhou, Guangdong 510900, China

## Abstract

Excitatory amino acid transporter 2 (EAAT2), also known as glial glutamate transporter type 1 (GLT-1), plays an important role in maintaining the extracellular glutamate concentrations below neurotoxic levels. The highly conserved TM2 transmembrane domain of GLT-1 maintains a stable position during the transport cycle; however, the effect of the transport cycle on the topology of TM4 in not well established. To further reveal the function of TM4, two cysteine pairs between TM2 and TM4 were introduced using site-directed mutagenesis. A significant decrease of transport activity was observed in the I93C/V241C and I97C/V241C mutants upon application of the oxidative cross-linking reagent, copper (II) (1,10-phenanthroline)_3_ (CuPh), which suggests that a conformational shift is essential for transporter activity. Furthermore, the decrease in activity by CuPh crosslinking was enhanced in external media with glutamate or potassium, which suggests that TM2 and TM4 assume closer proximity in the inward-facing conformation of the transporter. Our results suggest that the TM4 domain of GLT-1, and potentially other glutamate transporters, undergoes a complex conformational shift during substrate translocation, which involves an increase in the proximity of the TM2 and TM4 domains in the inward-facing conformation.

Glutamate is the most important excitatory neurotransmitter in the central nervous system. It must be immediately removed after it has been released into the synaptic cleft and transmitted a nerve impulse. Glutamate transporters, also referred to as excitatory amino acid transporters (EAATs), are responsible for the recycling of glutamate in the synaptic cleft, thus maintaining the external concentration of glutamate below neurotoxic levels and ensuring the precise control of excitatory synaptic transmission. Five mammalian subtypes of EAATs have been characterized so far: EAAT1–EAAT5^1^,^2^,^3^,^4^,^5^,^6^. The members of the glutamate transporter family, which include the EAATs, as well as prokaryotic transporters and two Na^+^-dependent neutral amino acid transporters, are about 25 to 30% identical^7^. This homology results in a similar membrane topology and transport mechanism for all family members^8^,^9^,^10^,^11^,^12^,^13^,^14^,^15^,^16^,^17^,^18^,^19^,^20^. The EAATs drive glutamate uptake by the cotransport of three sodium ions and one proton ion and the countertransport of one potassium ion^21^. The transport of the substrate against the concentration gradient by EAATs also uses energy, and this energy is generated indirectly by the Na^+^/K^+^-ATPase pathway^22^.

The crystal structures of prokaryotic aspartate transporter homologues in different states have been resolved^9^,^16^,^20^,^23^,^24^,^25^. The experimentally determined membrane topology of the glutamate transporter EAAT2 (also known as glial glutamate transporter type 1, GLT-1) is supported by the crystal structure of a homolog of eukaryotic glutamate transporters, Glt_Ph_^8^,^12^,^13^,^18^,^20^. The first resolved crystal structure of Glt_Ph_ revealed a bowl-shaped homotrimer with three identical monomers^20^. Every monomer functions as an independent unit with an individual substrate-binding site. Each monomer can be subdivided into two parts: the transporter core and the transporter cylinder. The transporter core contains the substrate and ion binding sites and is comprised of two reentrant helical hairpin loops (HP1 and HP2) and transmembrane (TM) segments 7 and 8. HP1 is speculated to form the internal gate of the transporter, while HP2 forms the external gate^9^,^10^,^16^,^17^,^20^,^26^. The transporter cylinder, which surrounds the transporter core, and maintains balance during the transport cycle, is comprised of TM1 through TM6^9^,^16^,^20^.

Previous experiments to characterize the spatial relationships between the tip of HP1 or HP2 and TM4 have suggested that TM4 may undergo a complex conformational shift during the transport cycle^27^. However, TM2, which is highly conserved among Glt_Ph_ and other transporter subtypes, is thought to maintain the balance of the transporter during the translocation of the transporter core^20^. Therefore, we speculated that we might obtain additional information about the conformational shift in TM4 during the transport cycle by investigating the effects of crosslinking TM4 to TM2. We introduced cysteine pairs into TM2 and TM4 of GLT-1 and examined the effects of the oxidative cross-linking reagent, copper (II) (1,10-phenanthroline)_3_ (CuPh) on transport activity. We also examined whether application of glutamate and potassium might have additional effect on the transport activity of the mutants. Moreover, we examined the aqueous accessibility of single cysteine mutants in various external media using the membrane-impermeable sulfhydryl reagent (2-trimethylammonium) methanethiosulfonate (MTSET). Our data suggest that there is a complex relative motion between TM2 and TM4 and that a conformational shift of TM4 may occur during substrate transport cycle.

## Results

### Inhibition of transport of cysteine mutants by CuPh

**Figure 1:**
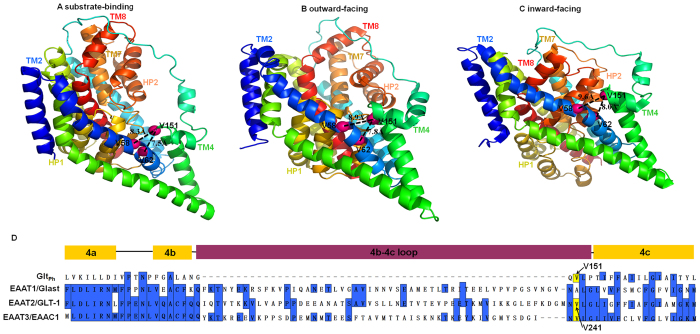
Membrane topology model of GLT-1/EAAT2. A schematic diagram is shown of the relative locations of Val-58, Val-62, and Val-151 of Glt_ph_ on TM2 (dark blue) and TM4 (green) in the substrate-binding (**A**) PDB ID: 1XFH), outward-facing (**B**) PDB ID: 2NWW) and inward-facing (**C**) PDB ID: 3KBC) structures. The three structures are viewed from the outside with dashed lines indicating the distances between the Cα-atoms (rose spheres) of the indicated residues. Val-58, Val-62, and Val-151 are equivalent to Ile-93, Ile-97, and Val-241 of CL-GLT-1, respectively. (**D**) Comparison of the amino acid sequences of TM4 between Glt_ph_ and EAATs. Regions of high homology of Glt_Ph_ and EAATs are highlighted in blue. The alignment of V151 of Glt_Ph_ and V241 of GLT1 is highlighted in yellow. The 4b,4c loop is present only in the EAAT transporters.

To determine the function of the TM4 domain of GLT-1, we constructed three double mutants for reversible cross-linking of the TM2 and TM4 domains, which are thought to lie in close proximity to each other. The Ile-93, Ile-97, or Leu-94 residues within the TM2 domain of cysteine-less GLT-1 (CL-GLT-1), together with the Val-241 residue within the TM4 domain, were mutated to cysteines to create I93C/V241C, I97C/V241C and L94C/V241C double mutants. While the molecular structure of GLT-1 has not been determined, structural models for Glt_Ph_, which is in the same family of transporters, show that it can assume substrate-binding, outward-facing or inward-facing conformations (Fig. 1A–C). The Val-241 residue of GLT-1, which aligns with the Val-151 residue of Glt_Ph_ (Fig. 1D), resides in the TM4 domain and is predicted to be close to TM2 amino acids in all three conformations.

**Figure 2:**
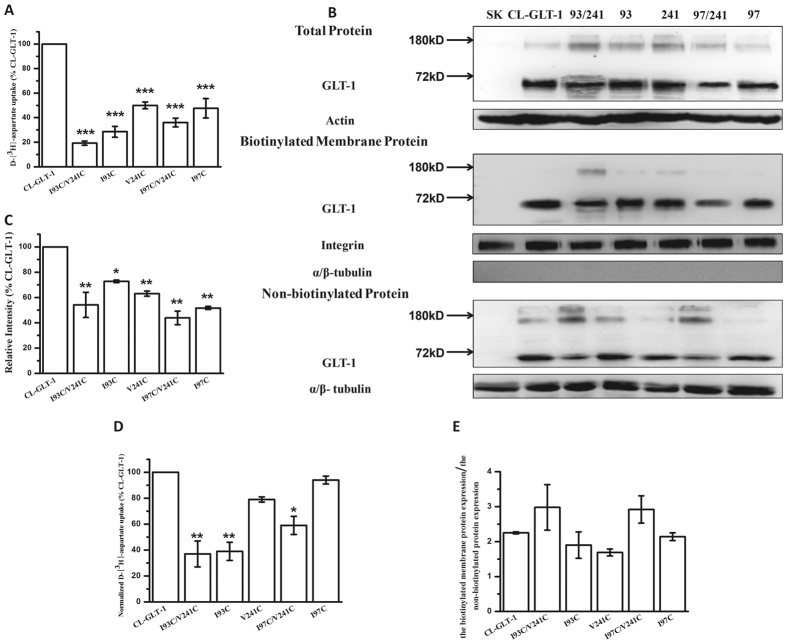
D-[^3^H]-Asp uptake activity and membrane expression of mutants. HeLa cells were transfected with the CL-GLT-1 or constructed mutants. (**A**) Transport activity of the mutants were measured in Hela cells as described under Materials and Methods and expressed as a percentage of CL-GLT-1. (**B**) The total proteins, biotinylated membrane proteins and non-biotinylated proteins were measured by western blot as described under Materials and Methods. Blots of all proteins were probed with the anti-GLT-1 antibody. Each blot of the biotinylated membrane proteins was probed for the internal plasma membrane marker -integrin and the absence of α/β-tubulin, an endogenous cytosolic protein representing the negative control in the biotinylated membrane proteins. Each blot of the non-biotinylated proteins was probed for the presence of α/β-tubulin. (**C**) Densitometric analysis of the biotinylated membrane proteins for each mutant normalized to internal marker (integrin) and represented as a percentage of CL-GLT-1. (**D**) D-[^3^H]-Asp uptake activity normalized to relative cell surface expression. (**E**) The ratio of the biotinylated membrane protein expression and non-biotinylated protein expression of CL-GLT-1 and mutants. Values represent the means ± S.E. of at least three different experiments done in triplicates. Values that are significantly different from that of CL-GLT-1 were determined by one-way ANOVA (*P < 0.05; **P < 0.01; ***P < 0.01).

The double mutants were expressed in HeLa cells, and initial experiments were performed to assess the effects of mutation on the transport of radiolabeled substrate in the absence of CuPh crosslinking (Fig. 2A). Our results showed that the protein expression of all the mutants were significant reduced comparied with CL-GLT-1 (Fig. 2B,C), and the transport function of I93C, I93C/V241C and I97C/V241C were impaired (Fig. 2D). In addition, comparing with CL-GLT-1, there was no difference for the ratio of the biotinylated signal and the non-biotinylated signal of each mutant, which indicated that the trafficking of the mutants to the plasma membrane are normal (Fig. 2E). We elected to compare transport activities with and without CuPh as an internal control for the individual activity of each protein. On the other hand, L94C/V241C showed more radically impaired substrate transport in the absence of CuPh (only 8.3 ± 2.3% activity), and consequently L94C/V241C was not used for further testing.

**Figure 3:**
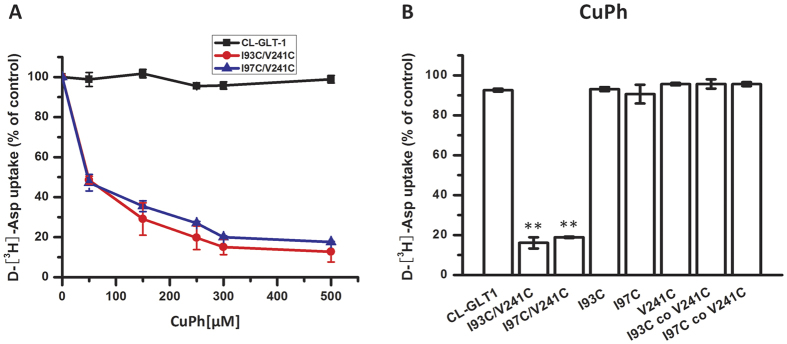
Inhibition of transport of cysteine mutants by CuPh. (**A**) Dose-response effect of CuPh on D-[^3^H]-Asp transport activity of CL-GLT-1, I93C/V241C and I97C/V241C. (**B**) Inhibition of transport by 300 μM CuPh is also shown for I93C/V241C and I97C/V241C double mutants, as well as for transporter proteins harboring corresponding individual mutants. The bars marked “I93C co V241C” and “I97C co V241C” represent individual mutants that were co-transfected. In each case, cells expressing mutants and CL-GLT-1 were treated with CuPh in NaCl solution for 5 min before the transport assay. Data represent the percentages of the remaining uptake activity of samples relative to those without any CuPh treatment (mean ± S.E., *n* = 3). Values that are significantly different from those of CL-GLT-1 were determined by one-way ANOVA (*P < 0.05; **P < 0.01).

Further examination of I93C/V241C and I97C/V241C demonstrated that substrate uptake was decreased by CuPh in a dose-dependent manner, though the transport activity of CL-GLT-1 was not significantly inhibited at a range of CuPh doses (Fig. 3A). This inhibition of transport activity of the double mutants may be due to the formation of a disulfide bond between the two introduced cysteines that blocks the conformational shift of the transporter^8^,^14^,^28^. About 50% inhibition of transport activity was observed at 50 μM for I93C/V241C and I97C/V241C while about 80% inhibition of transport activity was observed at 300 μM. Moreover, despite the significant inhibition of the double mutant, 300 μM CuPh did not cause inhibition in cells expressing single mutants that were either transfected individually or were co-transfected (Fig. 3B). These data indicate that the formation of a disulfide bond between two introduced cysteines within the same monomer is essential for CuPh-dependent inhibition.

The maximum transport rate (*V*_*max*_) and the apparent transport affinity (*K*_*m*_) of substrate were measured with or without pretreatment of CuPh to determine whether cross-linking changes the kinetic parameters. As shown in Table 1, both I93C/V241C and I97C/V241C showed a lower *V*_*max*_in the presence of CuPh. This indicates that the oxidative cross-linking between I93C and V241C or I97C and V241C damages transport function. In the absence of CuPh the *K*_*m*_ values of all of the mutants were comparable to that of CL-GLT-1; however, incubation of the I93C/V241C and I97C/V241C double mutants with CuPh caused a clear elevation in the *K*_*m*_ values of these double mutants, while the corresponding individual mutants showed no change. These results indicate that the formation of disulfide-bonds between TM2 and TM4 specifically impairs the substrate-binding ability of the GLT-1 transporter.

### The restorative effect of DTT on the CuPh-mediated inhibition of the transport activity of double mutants

**Figure 4:**
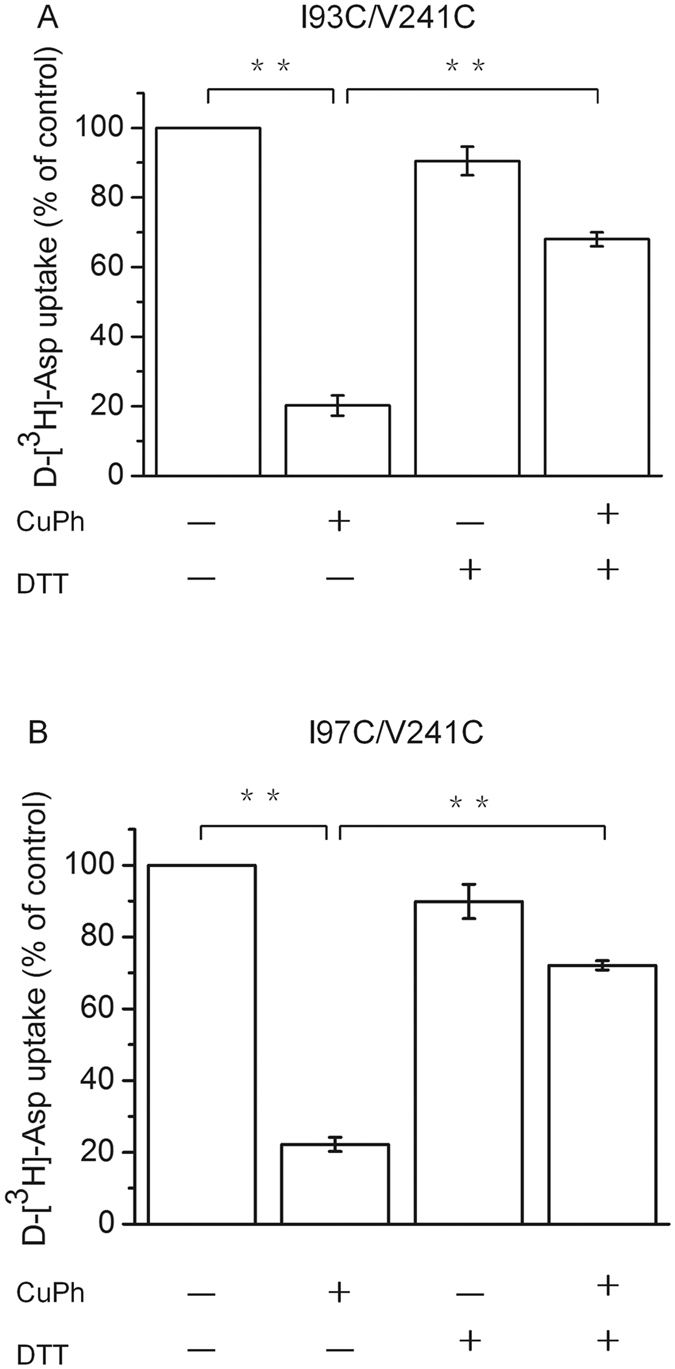
The restorative effect of DTT on the CuPh-mediated inhibition of double mutants. HeLa cells expressing I93C/V241C (**A**) or I97C/V241C (**B**) were preincubated for 5 min in the presence and absence of 20 mM DTT and then incubated with or without 300 μM CuPh and D-[^3^H]-Asp uptake was measured. Values are shown as a percentage of the uptake of mutants without CuPh and DTT treatment and represent means ± S.E for at least three independent experiments. Values that are significantly different from that of the control were determined by one-way ANOVA (*P < 0.05; **P < 0.01).

In rare cases, the formation of a covalent bond can occur between a sulfhydryl and another chemical group in the presence of CuPh. To rule out this possibility and to further verify the specificity of the inhibition by CuPh, we used the reducing agent dithiothreitol (DTT) to reverse the formation of the dicysteine sulfhydryl bond. Consistent with the above findings, the transport activities of I93C/V241C and I97C/V241C were inhibited to 21.2 ± 2.9% and 22.2 ± 2.0% by the application with 300 μM CuPh; however, simultaneous treatment with 20 mM DTT restored the transport activities of the double mutants to 68.0 ± 2.1% and 72.1 ± 1.3% (Fig. 4A,B). By comparison, DTT had no significant effect on the activity of CL-GLT1 (data not shown). These findings further confirm that CuPh induces the formation of the covalent bonds between the two cysteines that were introduced into the double mutants.

### Inhibition of transport of cysteine mutants by cadmium

**Figure 5:**
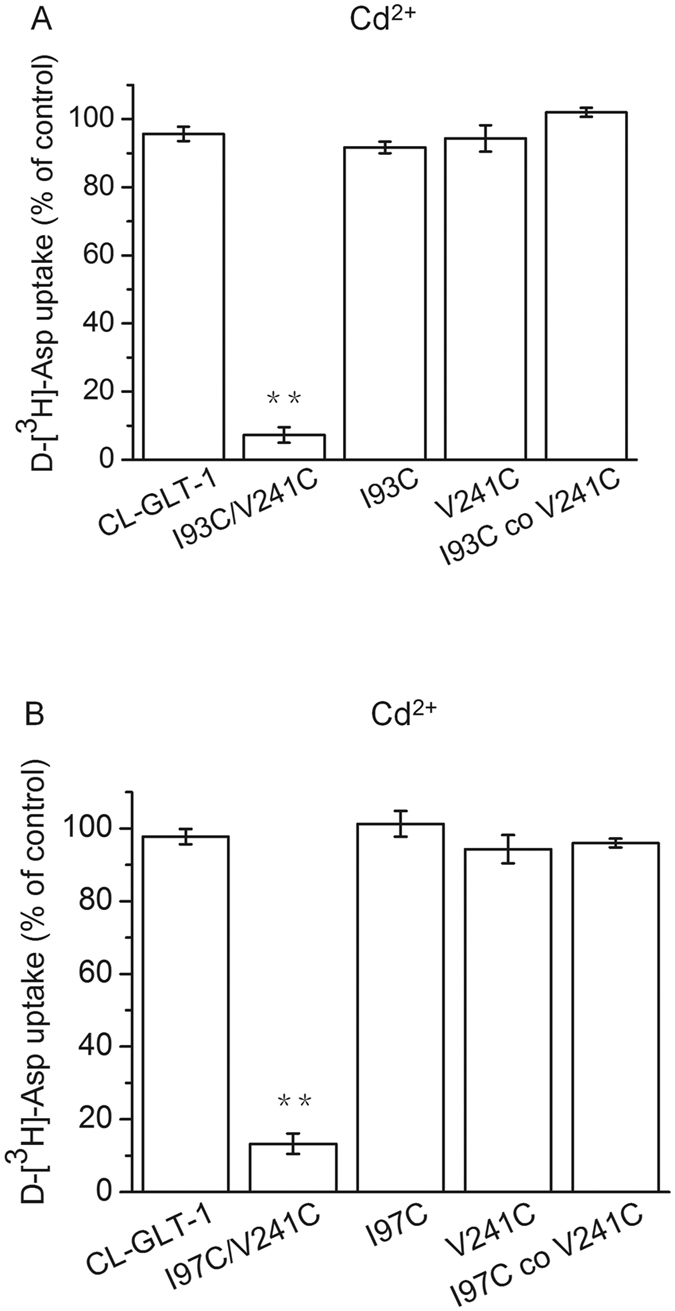
Inhibition of transport of cysteine mutants by cadmium. HeLa cells expressing I93C/V241C (**A**) or I97C/V241C (**B**) or the indicated controls were washed once with choline chloride-containing solution and assayed for transport in the presence or absence of 500 μM cadmium chloride. Values shown are the percentage of the activity in the presence relative to the absence of 500 μM cadmium chloride. Values represent the means ± S.E. of at least three different experiments done in triplicates. Values that are significantly different from that of CL-GLT-1 were determined by one-way ANOVA (*P < 0.05; **P < 0.01).

To verify that the two mutations for each of the double mutants are closely positioned within the GLT-1 protein, we assessed the effects of cadmium (Cd^2+^), which is known to bind the side chains of two coordinated cysteines with high affinity^29^,^30^. Substrate uptake was decreased after exposure to 500 μM Cd^2+^ for both I93C/V241C (7.3 ± 2.3% of control) and I97C/V241C (13.3 ± 2.8% of control). In contrast, cells expressing single mutants alone or in combination were not significantly affected by 500 μM Cd^2+^ (Fig. 5A,B). These data support the intramolecular spatial proximity of Ile-93 and Ile-97 in TM2 to Val-241 in TM4.

### Effect of external media on the CuPh-mediated inhibition of the transport activity of double mutants

**Figure 6:**
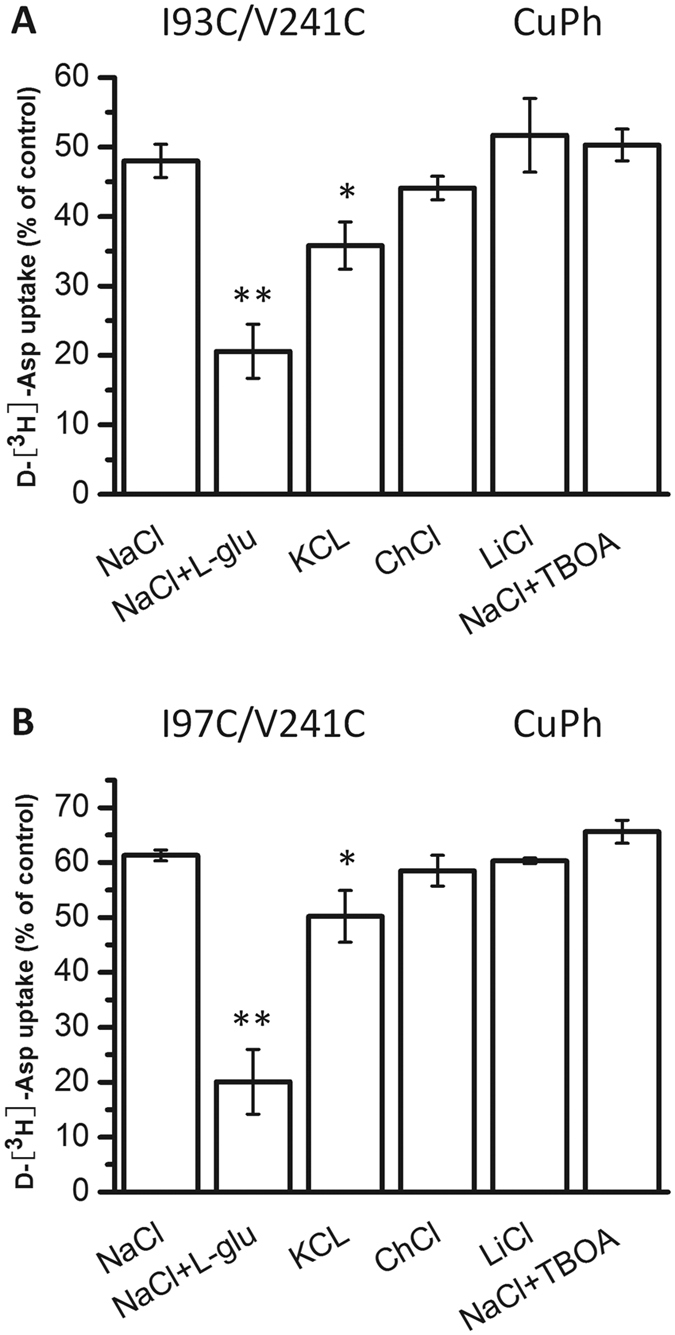
Effect of external media on the CuPh-mediated inhibition of the transport activity of double mutants. HeLa cells expressing I93C/V241C (**A**) or I97C/V241C (**B**) were preincubated for 5 min in external media containing one of the following: NaCl, NaCl + 1 mM L-glutamate, KCl, choline chloride, lithium chloride or NaCl + 20 μM TBOA. The cells were treated in the absence or presence of 50 μM CuPh, and then, D-[^3^H]-Asp uptake was measured. Values are shown as a percentage of the uptake of double cysteine mutants without CuPh treatment and represent means ± S.E for at least three independent experiments. Values that are significantly different from that of the control were determined by one-way ANOVA (*P < 0.05; **P < 0.01).

To determine the influence of various external media on the oxidative inhibitory effect of cross-linking TM2 and TM4, the substrate uptakes of I93C/V241C and I97C/V241C were measured after incubation for 5 min with 50 μM CuPh. This concentration induces half-maximal inhibition in NaCl (Fig. 3A). When the external sodium was substituted with potassium or supplemented with glutamate, each of which increases the proportion of inward-facing transporter^16^,^31^, CuPh on I93C/V241C and I97C/V241C caused more notable inhibition (Fig. 6A,B, first three bars). This suggests that cross-linking of TM2 to TM4 is more detrimental for proteins that are in an inward-facing conformation. As a control, this enhanced inhibition was not observed when sodium was replaced by choline or lithium (Fig. 6A,B, fourth and fifth bars). Additionally, when the external sodium was supplemented with the non-transportable substrate analogue TBOA, which increases the proportion of transporters in the outward-facing conformation^9^, the inhibitory effects of CuPh on I93C/V241C and I97C/V241C were not significantly changed (Fig. 6A,B, last bar). These results suggest that the inhibitory effects of cross-linking TM2 and TM4 are increased for inward-facing transporters, but not for outward-facing transporters.

### Effect of external media on the inhibition of transport activity by MTSET in single mutants

There are two potential explanations for why the inhibitory effect by CuPh on transporter activity might differ according to the external media that is used. First, the enhanced effects of CuPh for the inward facing conformation could potentially be explained by a smaller spatial distance between I93 or I97 and V241. This possibility would suggest a divergence from the structural model for Glt_Ph_ and could potentially be explained by the 53 additional amino acids present in the TM4 domain of GLT-1 (Fig. 1). Our findings are consistent with this possibility.

**Figure 7:**
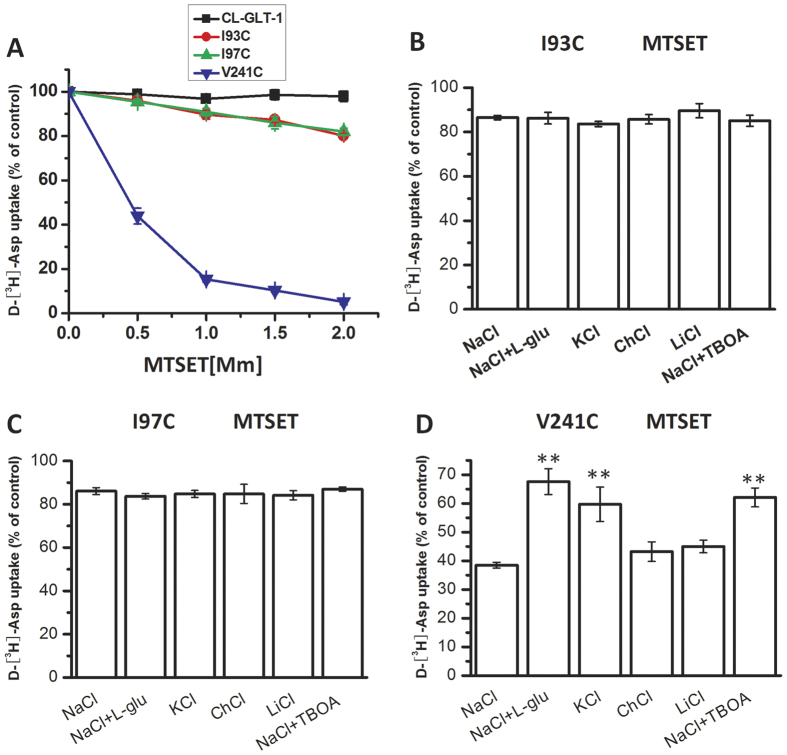
Effect of external media on the inhibition of transport activity by MTSET in single mutants. (**A**) Dose-response effects of MTSET on D-[^3^H]-Asp transport activity of CL-GLT-1, I93C, I97C and V241C are shown. HeLa cells expressing I93C (**B**), I97C (**C**), or V241C (**D**) were preincubated for 5 min in different external media containing one of the following: NaCl, NaCl + 1 mM L-glutamate, KCl, choline chloride, lithium chloride or NaCl + 20 μM TBOA. Cells were incubated in the presence and absence of 2.0 mM (**B,C**), or 0.5 mM (**D**) MTSET, and then, d-[^3^H]-aspartate uptake was measured. Values are shown as percentages of the uptake of double cysteine mutants without MTSET treatment and represent the means ± S.E for at least three independent experiments. Values that are significantly different from that of the control were determined by one-way ANOVA (*P < 0.05; **P < 0.01).

A second, less likely possibility is that the change in conformation of the transporter protein could affect the aqueous accessibility of CuPh to an individual cysteine residue. To rule out the latter possibility, the accessibility of each individual site was assessed using the membrane-impermeable sulfhydryl reagent MTSET. Our results demonstrate that the transport activity of a V241C single mutant decreased gradually with increased MTSET concentration; however, I93C and I97C were not sensitive to MTSET (Fig. 7A), either in NaCl media or in various other external media (Fig. 7B,C). After treatment with 0.5 mM MTSET, the transport activity of V241C was inhibited to about 40%, which is consistent with its aqueous accessibility within TM4. Furthermore, glutamate and external potassium partially reversed the MTSET-mediated inhibition of substrate uptake by V241C. A similar protection was also detected when external sodium was supplemented with TBOA (Fig. 7D). These results indicate that the change in accessibility of V241C is complex and cannot directly explain the increased CuPh inhibition of the I93C/V241C and I97C/V241C double mutants.

## Discussion

As the main transporters for clearing glutamate in the synaptic cleft, EAATs have been reported to be involved in the pathogenesis of various neurodegenerative diseases. Therefore, further research into the transport mechanism of EAATs is urgent. Though the EAATs are highly conserved and are similar in structure to Glt_Ph_, there are 53 more amino acid residues of TM4 in GLT-1 than in Glt_Ph_ (Fig. 1D). The differences in the number of amino acids between the TM4 of EAATs and Glt_Ph_ may lead to some differences in their spatial conformations and transport mechanisms^1^,^20^,^32^. Previous research investigating the spatial relationships between HP1 and TM4 or HP2 and TM4 suggests that TM4 may undergo a complex conformational shift during the transport cycle^27^. The aim of this work was to re-examine the conformational shift of TM4 during the substrate transport cycle in EAATs by investigating the proximity of residues in TM2 and TM4 in GLT-1.

We determined that CuPh can inhibit the transport activity of both I93C/V241C and I97C/V241C double cysteine mutants (Fig. 3), but that the addition of 20 mM DTT can reverse this effect (Fig. 4). Similar inhibition of transport activity was observed using Cd^2+^ (Fig. 5). We speculate that a disulfide bond between I93C/V241C or I97C/V241C forms upon incubation with CuPh or Cd^2+^ because of the close proximity between the residues Ile-93 and Val-241 or Ile-97 and Val-241. The formation of a disulfide bond would be presumed to lock the conformation of the transporter, which would obstruct further conformational shifts during the transport cycle. In the native molecule, the conformation of the transporter constantly changes during the substrate transport cycle. Our findings suggest that conformational lock leads to a decrease in transport activity.

We further examined the effects of conformational lock on transporters that were converted into predominate inward- or outward-facing conformation. The addition of the substrate glutamate or the replacement of sodium with potassium causes an increase in the percentage of inward-facing transporters^16^,^31^. Conversely, the substrate inhibitor TBOA increases the percentage of outward-facing transporters^9^. Therefore, we were able to observe the effects of CuPh cross-linking on different transporter conformations by modifying the medium outside of the cell. Our results show that the inhibitory activity of CuPh was further increased when the mutant transporters were converted to inward-facing conformations with either glutamate or potassium (Fig. 6). These data indicate that the distances between Ile-93 and Val-241 or Ile-97 and Val-241 become closer at the inward-facing state of the transporter. Moreover, the inhibitory effect of CuPh on I93C/V241C and I97C/V241C had no obvious change when the transporter was trapped in the outward-facing state (Fig. 6). Short-lived conformations could potentially result in cross-linking, and we therefore examined cross-linking for different conformations, including the substrate-binding, outward-facing and inward-facing structures. The results show that cross-linking can form at these three conformations, but that the decrease in activity by CuPh cross-linking was most severe at the inward-facing conformation. Therefore, it is likely the inward-facing conformation represents a naturally-occurring structure. These findings suggest a divergence from the findings for Glt_Ph_. According to the crystallographic structure of Glt_Ph_, the Cα-Cα distances of Val-58/ Val-62 and Val-151, which are equivalent to Ile-93/ Ile-97 and Val-241 of CL-GLT-1, are similar in the substrate-binding, outward-facing and inward-facing structures (8.3, 8.9 and 9.6 Å for Val-58 to Val-151; 7.5, 7.8 and 8.0 Å for Val-62 to Val-151; Fig. 1A–C). The TM4 domains of both Glt_Ph_ and EAATs contain three separated α-helixes: TM4a, TM4b and TM4c. However, Glt_Ph_ and the EAATs differ in the residues that separate these helixes: there are only 5 amino acid residues between TM4b and TM4c in Glt_Ph_, while there are 58 amino acid residues that form the TM4b-4c loop in GLT-1^20^. The extra 53 amino acid residues of TM4 in GLT-1 are likely to result in their differences in spatial conformation with a conformational shift that increases the proximity of TM2 and TM4 in the inward-facing conformation of the transporter.

The difference in the inhibitory effect by CuPh on transport activity could potentially be explained by changes in aqueous accessibility of individual cysteines. To assess this possibility, we treated the cells with MTSET, a membrane-impermeable sulfhydryl reagent that can interact with individual cysteines and form a disulfide bond that locks the transporter structure. I93C and I97C were not sensitive to MTSET. However, MTSET resulted in a dose-dependent reduction in substrate uptake of V241C (Fig. 7A). To detect changes in the aqueous accessibility of the single cysteine mutants, we added various external media. The inhibition of transport activity by MTSET did not differ for I93C and I97C in inward or outward-facing conformations (Fig. 7B,C). However, MTSET-mediated inhibition of transport activity by V241C was decreased in both the inward-facing and outward-facing states of the transporter (Fig. 7D). These experiments suggest that Val-241 is less accessible to the extracellular medium in the inward-facing and outward-facing state of transporter because of the conformational shift, which rules out a change in aqueous access as an explanation for the enhanced effects of applying CuPh in the presence of glutamate or potassium. Therefore, we infer that the inward-facing conformation leads to close spacing between TM2 and TM4 residues and that this shift in spacing is facilitated by the TM4b-c loop.

Though few research reports have focused on the TM4b-4c loop in EAATs, evidence supports the possibility that it has integral effects on EAAT activity. The TM4b-4c loop in GLT-1 has been demonstrated to contain a conformation-dependent trypsin cleavage site, suggesting that large conformational changes occur in this segment during substrate transport^33^. Furthermore, the TM4b-4c loop has been demonstrated to be exposed to extracellular solution^34^,^35^. The TM4b-4c loop extends from the center to the periphery of the transporter, and then back to the center^36^. In contrast to our findings, the results of fluorescence resonance energy transfer (FRET) suggest that there are no large conformational changes of the TM4b-4c loop during the transport cycle^36^. However, FRET reflects only the mean values of protein data in various transport states and therefore cannot indicate complex conformational changes. Using single molecule FRET to observe the motion of Glt_ph_ monomers, the conformational shift was shown to be random and independent^37^. A recent study has shown that anions can conduct conversion of transporters to an “outside” state by opening channels as a branching reaction from intermediate conformations. The lateral movement of transporter domains can promote instantaneous EAAT/Glt_ph_ anion channel opening^38^. Our studies showed that large changes occur for the distances between I93C (TM2) and V241C (TM4) or I97C (TM2) and V241C (TM4) during the transport cycle. Because the spatial position of TM2 remains unchanged during substrate uptake, large changes are likely to occur in the conformation of the TM4b-4c loop. The TM4b-4c loop may move inward with the transport core relative to the rest of the transporter, or it may rotate toward Ile-93 and Ile-97 in the inward-facing conformation of the transporter. Previous research showed that translocation of the fully loaded and occluded transport domain was affected by excision of the 3L4 loop^39^. According to our results, we suggest that the translocation failure of the transport core was due to the impaired inward-facing movement of TM4, which was caused by the removal of the 3L4 loop. In the outward-facing conformation of the transporter, which is induced by TBOA, the opening of HP2 causes no movement of the TM4b-4c loop. Furthermore, the TM4b-4c loop may have an effect on substrate transport in eukaryotic glutamate transporters. The TM4b-4c loop may play an importantly assistive role in the conformational changes of the transport channel because it can greatly support the conformational shift of HP1, HP2 and TM7 and promote substrate release into the cell. At the same time, specific amino acid residues of the TM4b-4c loop may be involved in the constitution of the transport channel. These hypotheses warrant further research.

## Materials and Methods

### Generation and subcloning of mutants

Site-directed mutagenesis was performed as previously described using DNA encoding a cysteine-less GLT-1 (CL-GLT-1) (from rats) as a template^40^,^41^. *Escherichia coli* CJ236 (dut^−^, ung^−^) was used for transformation. A single-stranded DNA molecule containing uridine was produced according to the experimental protocol from Stratagene with helper phage R408. Mutagenic antisense primers were used to introduce the mutations. The mutant segments were subcloned into the CL-GLT-1 sequence in the vector pBluescript SK (-) using the restriction enzymes *Eco*RI and *Avr*II or *Avr*II and *Xba*I. Finally, the mutagenesis and cloning of the constructs were confirmed by sequencing.

### Cell growth and expression

HeLa cells were purchased from ATCC (Manassas, VA, USA) and cultured in Dulbecco’s Modified Eagle’s Medium supplemented with 10% fetal calf serum, 200 U/ml penicillin, 200 μg/ml streptomycin, and 2 mM glutamine. Confluent HeLa cells in 24-well plates were infected with recombinant vaccinia/T7 virus vTF using 150 μL of virus in serum-free Dulbecco’s Modified Eagle’s Medium as previously described^42^. After 30 min incubation, the CL-GLT-1 or mutant constructs were transfected into HeLa cells using 1,2-dioleoyl-3-trimethylammonium-propane (DOTAP). Cells were incubated at 37 °C for 16–20 h prior to the transport assay.

### Transport assay

The uptake of d-[^3^H]-aspartic acid was measured after 16–20 h transfection. The wells were washed once with choline chloride (ChCl) solution (150 mM ChCl, 5 mM KPi, pH 7.4, 0.5 mM MgSO_4_, and 0.3 mM CaCl_2_) and then incubated with NaCl solution (150 mM NaCl, 5 mM KPi, pH 7.4, 0.5 mM MgSO_4_, and 0.3 mM CaCl_2_) supplemented with 0.4 μCi (0.15 μM) d-[^3^H]-aspartate for 10 min. Ice-cold NaCl solution was added to stop substrate uptake, and the cells were lysed with 1% sodium dodecyl sulfate. Finally, radiolabeled aspartic acid in the HeLa cells was assayed by liquid scintillation counting. All uptake data were presented after substracting the values obtained in cells transfected with the vector pBluescript SK (-).

### Cell surface Biotinylation

HeLa cells were plated onto 6-cm plates and after 24 hour cells were transfected with the CL-GLT-1 or mutant constructs. HeLa cell surface expression levels of CL-GLT-1 and the mutants were tested using the membrane-impermeable biotinylation reagent EZ-Link^TM^ Sulfo-NHS-SS-Biotion (Thermo Scientific, #21331). HeLa cells in 6-cm plates were washed twice with ice-cold PBS (pH 8.0) after 16–20 hours transfection and then incubated with 2.5 ml of EZ-Link^TM^ Sulfo-NHS-SS-Biotion (0.5 mg/ml in PBS) in two successive 20 minute incubations on ice with gentle shaking. The cells were washed twice with 100 mM glycine to removed non-reacted biotinylatioan reagent by incubation on ice for 20 min. The cells were lysed on ice for 20 min in 750 μl of cell lysis buffer. The cell debris was removed by centrifugation at 12,000 × *g* for 20 minute at 4 °C. Supernatants (the total proteins) were transferred to new tubes and 200 μl of streptavidin-agarose beads were added in order to bind the biotin-labeled cell membrane proteins. Centrifugation for 1 min at 4 °C and supernatants (the non-biotinylated proteins) were transferred to new tubes, then washing three times with ice cold lysis buffer. At last washing once with ice cold PBS and centrifugation for 1 min at 4 °C. The lower were the member protein samples (the biotinylated proteins) and the total proteins, non-biotinylated proteins were used for western blot.

### Western blot analysis

Samples were boiled at 55 °C for 30 min with protein loading buffer. Protein samples were fractionated by 10% SDS-PAGE, and then proteins were transferred onto polyvinylidene difluoride membranes. After blocking with 5% BSA for 1.5 h at room temperature, the membranes were incubated overnight at 4 °C with an anti-GLT-1 antibody. Integrin was used as an internal control. The membranes were washed three times with TBST and incubated with secondary antibodies for 1 h at room temperature. The different bands of protein were visualized with enhanced chemiluminescence by an imaging system. We converted the western blotting bands to the gray value using the software Image J.

The biotinylated membrane protein expression and the non-biotinylated protein expression were calculated as the optical density of monomer bands for CL-GLT-1 and its mutants in western blots divided by the corresponding value for integrin, α/β-tubulin respectively in individual samples. ^3^H labeled substrate uptake activity was normalized to relative cell surface expression^43^,^44^.

### Inhibition of transport by CuPh

Each well was washed once with ChCl solution. Then cells were incubated with different concentrations of CuPh for 5 min at room temperature. After 5 min, the medium was sequentially removed, and the cells were washed twice with the ChCl solution followed by the detection of transporter-mediated d-[^3^H]-aspartic acid uptake. CuPh stock solution was prepared by mixing 0.4 ml of 1.25 M 1,10-phenanthroline in water:ethanol (1:1) and 0.6 ml of 250 mM CuSO_4_. Each experiment was performed at least three times.

### Restoration of activity with dithiothreitol

Each well was washed once with ChCl solution. Then the cells were incubated with CuPh at the indicated concentrations for 5 min at room temperature. After 5 min, the medium was sequentially removed, and the cells were washed twice with the ChCl solution. Cells were preincubated with 20 mM dithiothreitol (DTT) for 5 min at room temperature, and subsequently were washed again with ChCl solution followed by the detection of transporter-mediated d-[^3^H]-aspartate uptake. Each experiment was performed at least three times.

### Inhibition of transport by Cd^2+^

Each well was washed once with choline solution. Then cells were incubated with 500 μM cadmium chloride in NaCl solution with d-[^3^H]-aspartic acid for 10 min at room temperature. After 10 min, the transport medium was removed sequentially, and the cells were washed twice with cold NaCl solution. Finally, cells were lysed with SDS followed by scintillation counting.

### Kinetics assay

Cells were incubated with 154 nM d-[^3^H]-aspartic acid combined with unlabeled d-aspartate at final concentrations of 1, 3, 10, 50, 100, 500, 1000, and 1500 μM for 10 min at room temperature after pretreatment in the absence or presence of CuPh at indicated concentrations. The *K*_*m*_ and *V*_*max*_values were measured with the Hill equation by using non-linear fitting in Origin 7.5 software (Northampton, MA, USA). The *V*_*max*_ was expressed as a percentage of the *V*_*max*_ of CL-GLT-1 without CuPh treatment. Each experiment was performed at least three times.

### Effects of external media on oxidative inhibition by CuPh or sulfhydryl reagent

Each well was washed once with ChCl solution. Then cells were incubated with media of different compositions with or without the indicated concentrations of CuPh or MTSET for 5 min at room temperature. The preincubation media contained one of the following: NaCl, NaCl + 1 mM l-glutamate, NaCl + 20 μM d,l-threo-β-benzyloxyaspartate (TBOA), KCl (150 mM KCl, 5 mM KP_i_, pH 7.4, 0.5 mM MgSO_4_, and 0.3 mM CaCl_2_), ChCl, or LiCl (150 mM LiCl, 5 mM KPi, pH 7.4, 0.5 mM MgSO_4_, and 0.3 mM CaCl_2_). After 5 min, the medium was sequentially removed, and the cells were washed twice with the ChCl solution followed by the detection of transporter-mediated d-[^3^H]-aspartate uptake. Each experiment was performed at least three times.

### Statistical analysis

All the data are presented as means ± SE from three independent experiments. One-way ANOVA with post-hoc multiple comparison or the Student’s *t* test was used to accomplish the statistical analyses with SPSS 16.0 statistical software. Differences were considered significant at *P* < 0.05 or *P* < 0.01.

## Additional Information

**How to cite this article**: Rong, X. *et al*. TM4 of the glutamate transporter GLT-1 experiences substrate-induced motion during the transport cycle. *Sci. Rep.* **6**, 34522; doi: 10.1038/srep34522 (2016).

## Figures and Tables

**Table 1:** Kinetic parameters of transporter mutants in the absence or presence of CuPh.

	*V*_*max*_	*K*_*m*_	**CuPh**	
			*V*_*max*_	*K*_*m*_
CL-GLT-1	100	31.3 ± 4.6	98.4 ± 1.2	30.7 ± 3.5
I93C/V241C	16.4 ± 1.2	29.2 ± 2.5	7.5 ± 1.3*	57.6 ± 2.7*
I97C/V241C	25.2 ± 2.3	34.1 ± 2.8	10.7 ± 1.6*	56.3 ± 2.4*
I93C	21.8 ± 2.6	29.8 ± 3.1	20.3 ± 2.2	31.1 ± 2.8
I97C	39.3 ± 2.1	30.6 ± 2.9	37.1 ± 2.5	29.5 ± 1.7
V241C	45.6 ± 3.4	28.3 ± 1.8	42.5 ± 1.6	30.6 ± 2.1

*V*_*max*_ and *K*_*m*_ values were calculated for CL-GLT-1, double cysteine mutants, and single cysteine mutants with or without 300 μM CuPh treatment. *V*_*max*_ is shown as a percentage of the *V*_*max*_ of CL-GLT-1 without CuPh treatment, while *K*_*m*_ is shown in micromolar units. Values are expressed as mean ± S.E for at least three independent experiments. Values that were significantly different in the absence and presence of CuPh, as determined by the Student *t*-test, are indicated (^*^P < 0.05).
